# Online propagation of emotions: A study of resharing dynamics on social media following celebrity suicides

**DOI:** 10.1371/journal.pone.0336134

**Published:** 2025-12-10

**Authors:** Ehsan Nouri, Nilesh Saraf, Jie Mein Goh, Srabana Dasgupta, Dianne Cyr

**Affiliations:** 1 Beedie School of Business, Simon Fraser University, Burnaby, British Columbia, Canada; 2 Karsh Institute of Democracy, University of Virginia, Charlottesville, Virginia, United States of America; University of Basel, SWITZERLAND

## Abstract

Emotional contagion on social media, particularly following shocking and tragic events, often unfolds through widespread resharing, amplifying affective responses that are typically intense and negative. This study focuses on the context of celebrity suicides, which have the potential to trigger emotional contagion and lead to adverse behavioral outcomes, such as copycat suicides. Using an exhaustive Twitter dataset covering four celebrity suicides, we theorize the propagation of emotional content through a valence-arousal framework, distinguishing emotions based on affective valence (positive or negative) and physiological arousal (high or low). We analyze how distinct emotions embedded in tweets propagate through retweet cascades, treating each tweet and its retweets as a single cascade. Propagation is measured across four cascade dimensions: size, lifetime, speed, and burstiness. Emotions are extracted from over a million tweets and retweets using a BERT-based language model and are used as predictors in regression analyses of the propagation metrics. Our results show that emotional messages propagate in distinct ways after tragic events. Disgust emerges as the most contagious emotion, spreading quickly, widely, and with longevity, while fear, despite its arousal, spreads weakly. Anger and surprise generate fast but short-lived cascades marked by high burstiness. Joy, though less frequent, endures longer than neutral and negative content, reflecting resilience but with lower burstiness. These findings advance research on online emotional propagation by demonstrating that discrete emotions differ significantly even within the same valence–arousal characteristic. They also offer insights for public health strategies to mitigate risks linked to emotional amplification in digital environments.

## Introduction

Online Social Media (SM) enables the widespread dissemination of user-generated content on an unprecedented scale [1–3]. This is facilitated by features such as resharing and algorithmic recommendations, which have transformed SM into a vital tool for communication and discourse, capable of shaping public opinion and driving behavior change [4,5]. Notable examples of SM’s influence include its role in mobilizing social movements such as the Arab Spring, Occupy Wall Street, and Black Lives Matter [6], as well as rallying citizens to provide aid during crises, such as natural disasters [7]. The remarkable power of SM to galvanize such large-scale responses among individuals with often tenuous connections lies not only in the informational value of the messaging but also in their emotional resonance [8,9]. In other words, SM can act as a powerful channel for emotional transmission, capable of radically influencing opinions and behaviors across online populations.

While examples such as protest mobilizations or citizen-led disaster responses highlight the positive impact of SM on society, its darker side is equally evident in the rapid spread of negative emotional content through these networks [8,10,11]. This is particularly concerning given that SM exposes users from diverse backgrounds to online content on a global scale, with exposure to negative emotions shown to adversely affect mental well-being, mood and, in extreme cases, trigger self-harm [12,13]. Research also indicates that negative discourse tends to grow faster and dominate SM platforms, overshadowing positive interactions, particularly after shocks and crises [11,14]. Moreover, people are generally more influenced by negative information than positive [10,15,16], a phenomenon known as the negativity bias. This bias, well documented across various contexts, even before the online era [17], is likely amplified in the digital age due to the scale and reach of SM platforms. Although the precise theoretical mechanism by which negative SM content shapes users’ feelings, opinions, and behavior remains unclear, mounting evidence suggests that contagion processes are at work, where repeated exposure to the same emotional content triggers online users to adopt the emotions and retransmit to others [18–20]. For example, users exposed predominantly to negative content tend to create and share more negative posts [10], creating a feedback loop that propagates negativity across the network [21]. This multiplier effect can cascade through the population, with its magnitude influenced by factors such as the context and connectivity of the networked community. Thus, research suggests that the online spread of negative content provides indicative—though not conclusive—evidence that emotion, not just information, is retransmitted online.

This study seeks to understand how the retransmission (resharing) of online emotional content unfolds on SM in the aftermath of high-profile tragic events that predominantly trigger negative emotions. Our motivation arises from the concerning, well-documented phenomenon that media coverage of suicides, particularly those involving prominent figures, can lead to copycat behavior in the broader population [22–26] —the “Werther Effect.” While prior research acknowledges the role of social media in spreading information and emotions [10,19,27,28], its mediating role in enabling copycat behavior through the distribution of emotions remains less understood, particularly whether tragic events trigger distinct *propagation* dynamics (used interchangeably with *diffusion*; see conceptual clarification in S1 Appendix). We therefore examine the temporal patterns of emotion propagation following tragedies, highlighting that each emotion follows its own trajectory and affects vulnerable audiences differently. This underscores the need for tailored intervention strategies that account for these dynamics. A deeper understanding of such patterns is critical to addressing the rapid and unpredictable effects of a fatal phenomenon that has become increasingly prominent in the digital age [22,23,29,30].

Hence, we pose the following research question: *(RQ) How does the retransmission of emotions expressed on social media platforms unfold in the aftermath of celebrity suicides?* To address this question, we analyze the emotional content of social media posts following multiple celebrity suicides and compare their “reshare cascades” across four distinct metrics of propagation. Similar to some of the previous studies [27], while focusing on reshare cascades allows us to apply current understanding of emotions, at the same time we also control for author level characteristics of the originating posts among other factors. By correlating the emotions in posts with multiple propagation (diffusion) metrics, we derive insights into how specific emotions shape the spread of content.

Our study advances understanding of suicide contagion by identifying emotional propagation as a potential mediating mechanism through which emotional and high-risk messages can reach vulnerable users and increase the likelihood of copycat suicides [22,23,29]. Next, by applying the valence–arousal framework, i.e., the circumplex model of affect [31] to celebrity suicides, we show that emotions with the same valence and arousal can still propagate very differently. Among high-arousal negative emotions, disgust spreads quickly and extensively with persistence, anger spreads rapidly but fades quickly, and fear shows weak propagation overall. By contrast, joy, a low-arousal positive emotion, endures longer than negative emotions despite spreading more slowly, reflecting resilience in collective responses. This evidence demonstrates that discrete emotions provide sharper insights than broad valence–arousal categories for understanding how emotional content circulates in highly visible tragedies [28,32].

Second, we demonstrate that propagation must be understood multidimensionally, rather than reduced to a single measure of virality. Prior studies often conflate metrics such as reshare volume, speed, and lifetime, treating them as interchangeable, yet our results show that they capture distinct dynamics. An emotion may spread with high speed but have only a short lifetime (anger), or diffuse more slowly yet endure longer despite weaker reach (joy).This challenges the prevailing assumption that high-arousal negative emotions always dominate diffusion [33], revealing instead that different emotions leave different propagation signatures across speed, virality, lifetime, and burstiness. Finally, we contribute methodologically by applying a fine-tuned BERT-based model [34] to detect discrete emotions in large-scale textual data, offering greater accuracy than lexicon-based approaches. Taken together, these contributions provide a richer theoretical and methodological foundation for studying emotional propagation in crisis contexts, situating celebrity suicides within broader models of social media–enabled health behaviors.

Our empirical setting and dataset offer several advantages. First, the unexpected nature of celebrity suicides minimizes the likelihood of strategic behavior by online actors, reducing the risk of artificially influenced or contaminated activity. Second, these events’ tragic and emotionally unambiguous nature limits interpretive variability across users—unlike political content, which often invites various speculation—enabling a clearer analysis of how emotions influence collectives. Third, the dataset predates the widespread use of advanced chatbots, avoiding complications introduced by automated activity in more recent data. Finally, at the time of data collection, suicide prevention and content filtering measures were limited, allowing for a more unobstructed view of organic emotional propagation on the platform.

The following sections begin with a review of the literature on emotional propagation in social media and its links to the distribution of self-harm behaviors. We then present our methodology for analyzing over a million tweets and retweets related to four celebrity suicides. Focusing on the temporal characteristics of retweet cascades—each comprising an original tweet and its reshares—we apply the valence-arousal framework [31] to theorize how the nature of emotions shapes individual responses and drives interpersonal propagation dynamics. Our analysis examines propagation across four key dimensions: volume, longevity, speed, and burstiness. This approach reveals how emotional content spreads in contexts dominated by negative emotions. We conclude by discussing the theoretical and practical implications of our findings for public policy, information systems, and related fields. Although our data predates recent platform changes, it provides enduring insights into the dynamics of emotional propagation and adds empirical support to recent work showing that distinct emotions follow distinct patterns of spread [28,32], which we identify through a multidimensional lens.

## Literature overview

### Social media and online content diffusion

Social Media enables the spread of user-generated content through the interconnected network of its users [3]. As content is reshared by users and distributed by platform algorithms, it can diffuse across the network, reaching a larger online population compared to the author’s primary contacts. This diffusion capability has attracted considerable interest across various research disciplines. In marketing, studies examine the role of SM in driving word-of-mouth promotion for products [35]. Political science and communication scholars have examined the spread of fake news and rumors on SM platforms [1,36–38]. Similarly, in the health sciences, SM is a critical tool for disseminating health behaviors and gauging public opinions and health choices during events such as pandemics [15,39]. Lastly, network scientists have also focused on understanding the underlying structures that enable information and behaviors to propagate through the interconnected links of social networks on SM platforms [3,40].

Research on the diffusion phenomenon of SM typically addresses three key aspects: structural features, temporal dynamics, and content characteristics [2]. First, scholars have emphasized the importance of social network structures formed through various connections between users, such as friendships, followers, group memberships, and resharing links. Structural features like user centrality, the presence of weak ties, and the formation of clusters play a critical role in shaping the outcomes of information diffusion [3,41]. Second, research has explored the temporal dynamics of diffusion events [36,38,42–44], focusing on the speed and duration of information spread. Key findings indicate that sharing behavior often occurs in clustered bursts and that the aggregate temporal patterns of communication vary depending on factors such as the topic [45], the emotions expressed [27,46,47], or the users involved [48]. Understanding these temporal patterns is crucial for decision-makers, including business managers, law enforcement, and crisis response teams, as it enables them to respond with effective intervention. Finally, the content of SM communications, ranging from text [33] to multimedia like videos [49] and emojis [50,51], has also been analyzed to examine how different forms of information, emotions, and meaning influence recipients. These studies highlight the differential impact of content characteristics on how information spreads and resonates with audiences.

To better understand the structural and temporal dynamics of information spread on social media, researchers often use the concept of cascade trees to model the propagation process through interpersonal transmission and resharing [1,36]. In this framework, the original post is the root of a tree-like graph that expands as users reshare it. Each node represents a user, and each branch corresponds to a resharing event. For instance, a chain of retweets on X (formerly Twitter) can be visualized as a cascade tree, illustrating who retweeted whom through added nodes and connections. These models are instrumental in analyzing diffusion patterns, including virality, influence, and the identification of key spreaders.

### Online propagation of emotions

Human emotions involve coordinated changes in perception, physiological responses, motivation, behavior, and feelings triggered by how individuals appraise events that matter to them [52]. Since external factors can trigger emotions in individuals, the emotional expressions in online content can act as powerful cues that stimulate users to mirror, amplify, or retransmit the emotion to others. As per the emotional contagion theory, the transfer of emotions can be automatic, unconscious and difficult to control, the primary pathways to such transfer are empathy which induces mimicry [53]. Further, research has also shown that online content can also reflect the emotions of its authors and serves as a pathway for transmitting emotions between users [21,54], even via mediums like emails and webpages [33], or through emojis [51]. This dynamic makes human collectives conducive to the emergence and propagation of emotional cascades, where the transfer of emotions spreads rapidly from a few individuals to a significant portion of a collective [21]. Such findings within online settings are significant because emotional transmission, once dependent on face-to-face cues like body language and vocal tones, has evolved to operate through computer-mediated communication and network-driven cues in online environments.

Research on the emotionality of SM content has evolved in two key directions. One foundational area is focused on the empirical evidence of emotional contagion, which differs from information diffusion. Emotional contagion occurs when content expressing a particular emotional state created by one user on SM invokes similar emotions in the recipients. For instance, Ferrara and Yang [10] show that exposure to negative (positive) content increases the likelihood of users posting negative (positive) content on Twitter. Similarly, in a large-scale Facebook experiment, Kramer et al. [19] found that emotional expressions in users’ news feeds influenced the emotions expressed in their subsequent posts. Their study also revealed that emotional exposure can impact social engagement, shaping user activity levels on the platform. Context also plays a significant role, as shown by Coviello et al. [54], who found that real-world events, such as rainfall, can predict online emotional responses. Notably, the emotions elicited by rainfall in one geographic location spread online to users in unaffected regions, highlighting the reach and influence of emotional contagion in online environments. However, asserting the underlying mechanism of contagion requires a deeper study of the latent psychological states of the users, either through recording subsequent posts or through experimental methods.

A related research stream shows that different emotions exhibit distinct diffusion or propagation patterns. For instance, a study on Weibo found that anger is more likely to spread between users than joy, whereas sadness has a weaker correlation among users [55]. Stieglitz and Dang-Xuan [8] observed that political tweets that are more emotionally charged get retweeted more frequently and at a faster pace. Similarly, analyzing thousands of rumors on Twitter, Pröllochs et al. [27] compared the diffusion rates of various emotion categories. They found that, unlike the emotions of surprise, fear, and disgust, expressions of anger, anticipation, and trust result in more reshares, reach a broader audience, and persist over a longer period. Berger and Milkman [33] also support these findings, showing that New York Times articles evoking high-arousal emotions, such as awe, anger, and anxiety, are more likely to go viral than those associated with low-arousal emotions, such as sadness.

The study of how emotions spread across social media platforms emphasizes the need for theoretical frameworks to comprehensively understand emotional propagation. Building on extant research, we draw from two foundational theoretical approaches that offer distinct but complementary perspectives to guide our analysis. First, Goldenberg and Gross [21] propose studying emotional contagion through an “emotional cascade approach,” which examines how a piece of content elicits sequential reactions or reshares after it is viewed by others. We adopt this framework to analyze reshares on Twitter in the aftermath of celebrity suicides. Second, we guide our empirical analysis using the “circumplex model of affect” [31], which categorizes emotions along two bipolar dimensions: valence (positive vs. negative) and arousal (the level of physiological activation they evoke). High-arousal negative emotions include anger, fear, frustration, distress, and annoyance, whereas low-arousal negative emotions encompass misery, sadness, and boredom. On the positive valence side, high-arousal emotions include happiness and excitement, while low-arousal positive emotions include satisfaction, joy, and relaxation. The model is a structure of the affective experience of individuals where emotions at the opposite end of each dimension seldom are experienced together. Thus, sadness and joy, which lie along the dimension of valence, do not typically occur together, or sadness and anger, which lie apart on the arousal dimension, seldom will be observed together.

Previous research on the online dynamics of emotional content has shown that high arousal and negative emotions are significant drivers of virality [28,33]. However, many studies examine arousal and valence separately and often restrict their analysis to a single metric of retransmission, with the exception of a few studies [10,27,33]. For example, while an existing study finds that emotions like fear and anger [55] are more likely to be reshared on social media, it does not examine other aspects of the temporal dynamics, such as the duration or speed with which the retransmission occurs. Overall, there is limited evidence in social media research distinguishing the temporal dynamics of diffusion created by various emotions. Recent studies that challenge valence–arousal frameworks highlight the potential of focusing on discrete emotions [28,32], and there is room to extend their findings by demonstrating how discrete emotions vary in their temporal patterns of diffusion.

### Self-harm and digital social influence

The third body of work informing our study pertains to suicide contagion, a phenomenon extensively studied across multiple disciplines, including health sciences, communication, and psychology. The literature widely recognizes suicide as a contagious act, with contagion occurring through proximity, demographic similarity, or the prominence of the victims. Instances of suicide contagion have been documented for at least two centuries [30,56]. Celebrity suicides, in particular, have been associated with inducing copycat behavior in the population as far back as the 18th century [24]. This effect is often facilitated by media exposure and cognitive mechanisms such as imitation and suggestion [57,58]. Research has shown that media publicity, even before the advent of digital and social media, played a significant role in triggering copycat suicides [26]. This phenomenon, commonly known as “The Werther Effect,” was first identified following the publication of Goethe’s novel “The Sorrows of Young Werther” [59], which reportedly led to a wave of suicides across Europe. Empirical evidence for this effect was later confirmed by Phillips [24], who analyzed suicides reported in newspapers in the US and UK and correlated them with subsequent increases in suicide rates within those communities.

More recent studies have demonstrated evidence of suicide contagion across different regions, including US, Europe, and East Asia [22,23,26,57]. For example, a study found that in August 2014, following the suicide of Robin Williams (celebrity actor), there was an excess of 1,841 monthly suicide cases in the US—a 10% increase over baseline rates [22]. Additionally, research on ethnic and locally clustered suicide events also suggests that individuals who are in contact with or associated with someone who has died by suicide are more likely to engage in the same act [60]. This subset of the more general realm of behavioral contagion underscores the urgency of preventive interventions, particularly in the early stages of a contagion event, when the spread can escalate rapidly. Special attention is needed for vulnerable populations, including youth and individuals with mental health conditions [61], who are more susceptible to social influence through exposure to others’ behaviors, emotions, and thoughts.

Social media expands interpersonal communication, potentially amplifying the extent of suicidal contagion. Content shared on SM following celebrity suicides often exhibits distinct emotional patterns. For instance, a study of Reddit posts following the suicide of ten celebrities found an increased expression of inward-focused content, anxiety, anger, and negative emotions [62]. Similarly, Niederkrotenthaler et al. [63] analyzed Twitter activity following the suicide of Swedish DJ Tim Bergling (Avicii), identifying several short-lived peaks of social media engagement appearing after the incident. Each wave of engagement aligned with major news revelations—initial death reports, confirmation of suicide, and disclosure of the method—highlighting how different types of information trigger distinct emotional and temporal patterns. Their analysis also revealed that tweets referencing the suicide method, though limited in volume, reached disproportionately larger audiences due to being shared by highly followed accounts. Fahey et al. [25] further examined the temporal evolution of emotions following celebrity suicides, showing that suicides of younger celebrities, women, and individuals in the entertainment industry tend to evoke stronger emotional responses. These emotional and textual dynamics are thought to be fundamental mechanisms through which exposure to suicide news and suicidal ideas occurs in digital environments, potentially triggering emotional or even behavioral contagion.

Social media also provides a valuable lens for understanding both the mental health of individuals and the mechanisms of social influence. Patterns in language, emotional tone, and engagement have been used to detect suicidal ideation and predict broader trends, including national suicide rates [62,64,65]. Distinct linguistic markers such as first-person pronouns, present-tense verbs, angry tones, and references to death are more prevalent in suicidal posts [66], revealing how online expression reflects and shapes collective responses to suicide-related events.

Despite these advances, there is a need for a deeper understanding of resharing—a core mechanism that can activate contagion—since it allows us to study the bottom-up development of cascades, thereby disseminating content and emotions to a larger audience. Earlier studies analyzed aggregate-level temporal patterns of tweets and their reply networks after celebrity suicides [63], as well as the emotional trends of communications classified by the characteristics of the deceased celebrity [25]. We suggest that the underlying temporal dynamics between individual retweets, as well as the cascade-level characteristics they generate (and how cascades vary in outcomes given their authors and content) will help us gain a deeper understanding of the potential micro-level mechanisms at play.

### Metrics of online propagation

The propagation (diffusion) of online content has been examined through multiple measures that collectively offer the potential to understand the underlying micro-level mechanisms. We revisit these approaches to construct a more integrated theoretical lens connecting tweet content to retransmission patterns. A primary measure is cascade *size*, which is widely used as a core indicator of content virality and influence. It refers to the total number of retweets (i.e., the number of nodes in a cascade) and serves as a dependent variable in many studies [8,36,67,68]. While simple and intuitive, this measure overlooks the temporal dynamics of diffusion. Del Vicario et al. [36] used cascade size to track misinformation spread, while Stieglitz and Dang-Xuan [8] applied it to compare the diffusion of emotional and non-emotional content. Juul and Ugander [71] further showed that cascade size explains much of the variance in how true and false news diffuses, even when controlling for structural and temporal differences.

Second, the *lifetime* of the cascade is defined as the time between the first and last event in the sequence of retweets, i.e., the time between the original tweet and the last retweet [36]. A cascade with a long lifetime implies that it has been reshared numerous times or that there have been long intervals when it was not reshared but was eventually. Overall, long lifetimes mean that the Tweet’s emotional content has remained visible to the population over a longer period [27,38]. It is also possible that cascade size and lifetime will be correlated within a population because exposure of a tweet to a larger number of users will increase the likelihood that it will continue being reshared over a longer period. Third, the *time-to-Nth* retweet refers to how long a cascade takes to reach a predefined growth threshold, such as the time to its 100th retweet [47]. This metric allows for comparing the diffusion speed across cascades while controlling for a baseline level of virality [8,47].

Finally, the burstiness of a cascade captures the excitability of online content. It reflects how reactions cluster in time, with many users resharing within a short span followed by periods of lower activity, indicating synchronized collective responsiveness rather than steady diffusion. In recent work, “bursts” of online activity observed in email communications suggest a self-excitement process [69]. Thus, each cascade essentially is a sequence of bursts where two cascades can have the exact same number of retweets, but one may have a greater number of bursts (irregularities) than the other. Arranging retweets within each cascade chronologically can help discern multiple retweet bursts, with each burst temporally distinguishable from adjacent bursts. The theoretical distinction between the various dependent variables in the literature provides a basis for understanding the nuances of how tweets are retransmitted differently depending on the dominant emotion they convey.

## Theoretical framework and hypotheses

This section outlines the theoretical basis for our empirical analysis, focusing on the relationship between emotional content in social media messages and their temporal patterns of propagation. We draw on Russell’s Circumplex Model of Affect [31], which maps emotions along arousal (high to low) and valence (positive to negative), to explain how these emotional qualities trigger cognitive responses at the individual level that may shape collective retransmission behavior. Prior experimental and quasi-experimental evidence shows that both arousal and valence influence sharing and resharing intentions [10,19,33]. High-arousal and negative emotions often exert stronger effects because they heighten mobilization and action tendencies, elicit mimicry and empathic alignment, and reflect the well-established negativity bias [17,53]. These dynamics are further amplified in digital environments where scaled exposure and cascading visibility accelerate diffusion [21]. Yet the empirical record remains fragmented, as most studies examine only one of the valence or arousal dimensions, focus on discrete emotions, or rely on limited of propagation. Our study addresses this gap by analyzing emotions through a valence–arousal lens across four propagation metrics: size, lifetime, speed, and burstiness, providing a more comprehensive account of how emotional content spreads in the aftermath of celebrity suicides.

We also consider the salience of our empirical context of self-harm by celebrities in our theorizing. In particular, we consider how the level of heterogeneity within the collectives, driven by subjective interpretations and varied emotional responses of the audience in our specific context, can generate propagation dynamics distinct from those in other studied contexts. However, the empirical context of self-harm skews SM content towards negative valence and thus precludes a full comparison with neutral and positive valence content. Therefore, all except one of our hypotheses are centered on the dimension of (high) arousal, with the one on (negative) valence.

### Arousal and cascade size

Cascade size, defined as the total number of users who reshare a message, is a widely used indicator for evaluating the spread of online content, particularly on social media. Prior research has shown that exposure to content containing high-arousal emotions—both positive (e.g., excitement, enthusiasm) and negative (e.g., anger, fear)—tends to generate greater mobilization and action-oriented responses, compared to low-arousal or neutral content [33,70,71]. For instance, Berger and Milkman [33] found that high-arousal emotions significantly increased the likelihood of New York Times articles being forwarded via email, an early but robust indicator of emotional virality.

On social media, this arousal-driven engagement translates directly into greater resharing probabilities, thereby increasing cascade growth. Each retweet amplifies the message’s visibility and increases its chances of further propagation by mobilizing emotionally stimulated audiences. This effect is well-documented in political messaging, where morally charged and emotionally provocative content often produces large cascades [8,72]; however, the underlying mechanism—high arousal driving user engagement and resharing—is broadly applicable across a variety of social media contexts. Even studies that do not categorize emotions using dimensional models [27,55], such as the valence–arousal framework [31], have observed similar patterns. In other words, regardless of their classification, tweets expressing high-arousal emotional states tended to produce larger cascade sizes. Thus, we propose our first hypothesis:

*Hypothesis 1: Social media messages containing high-arousal emotions receive a greater number of reshares than messages containing neutral or low-arousal emotions.*

### Arousal and cascade lifetime

The influence of emotional arousal on cascade lifetime is not consistent across social media contexts, and we argue that the emotional structure of the topic itself fundamentally shapes this variation. For instance, in emotionally ambiguous or open-ended contexts like online rumors, Pröllochs et al. [27] found that high-arousal emotions such as anger or anticipation prolong cascade lifetime; however, fear, despite being a high-arousal emotion, was linked to shorter lifetimes. Brady et al. [72] find that the effect of anger on social transmission varies across moral topics, such as climate change vs. same-sex marriage. These mixed findings suggest that emotional arousal alone may not consistently predict cascade longevity, likely due to the topic’s capability (e.g., rumors) to invite further speculation, reinterpretation, and debate from a heterogeneous and ideologically diverse user base or, conversely, trigger avoidance in the audience.

By contrast, celebrity suicides are emotionally unambiguous, tragic, and time-bounded events. They typically elicit rapid and high-arousal emotional responses such as grief, shock, and outrage, particularly from users with parasocial ties or cultural attachments to the celebrity. However, these emotionally engaged audiences tend to be affectively homogeneous—they share similar emotional states and interpretations—at least towards such tragedies. This emotional homogeneity fosters synchronized emotional reactivity, where users collectively engage in a brief but intense expression of emotion. While this initial surge drives early resharing, it saturates the finite pool of engaged users. Therefore, their emotional energy is exhausted, and no new interpretive frames emerge to sustain discussion, unlike rumors [27], which may invite retrospection.

Research in emotional contagion supports this pattern: homogeneous groups are more likely to experience strong but short-lived amplification as their emotional responses converge and saturate quickly [21,36,73]. Once this initial group has reshared the content, often within hours, the pool of emotionally susceptible users contracts rapidly. Combined with algorithmic prioritization of recency and a lack of ongoing narrative development, this leads to a steep drop-off in visibility and engagement. As a result, even though high-arousal emotional content spreads quickly, it tends to follow a burst–decay trajectory characterized by rapid saturation and a shorter cascade lifetime overall [63,74].

Therefore, while high-arousal emotions enhance the initial probability of resharing, we propose that in emotionally constrained contexts like celebrity suicides, such emotions lead to shorter, not longer, cascade lifetimes.

*Hypothesis 2: Social media messages containing high-arousal emotions are reshared for a shorter period than messages containing neutral or low-arousal emotions.*

### Valence and cascade lifetime

The relationship between emotional valence —particularly negative emotions— and cascade lifetime remains theoretically underdeveloped and empirically inconsistent. While substantial literature confirms that negative content increases engagement and resharing probability [8,10,43], this does not imply that such content persists longer. Unlike virality or reach, cascade’s lifetime reflects how long a message remains actively reshared in a network. In theory, a stronger emotional reaction to negative content could either *shorten* lifetime by driving a rapid saturation of engaged users or *extend* it by sustaining ongoing relevance and engagement across a broader user base.

Importantly, the empirical context can moderate how valence influences cascade dynamics. In ambiguous or ideologically contested contexts such as rumors and moral debates, negative emotions like anger can prolong cascade lifetimes by fueling speculation, reinterpretation, and ideological conflict [27,72]. In contrast, in unambiguous and tragic events like celebrity suicides, emotional responses are highly synchronized among affectively homogeneous communities that share grief, sadness, or shock. Research in emotional contagion and network homophily suggests that such alignment leads to strong but short-lived amplification, as the pool of emotionally susceptible users is quickly saturated [21,73]. Burnap et al. [47] observed similar dynamics following the Woolwich terrorist attack, where negative posts spiked and declined within a short window, indicating a brief lifespan. Moreover, algorithmic exposure mechanisms compound this trajectory. Once a burst of resharing subsides, content loses visibility due to recency decay and reduced engagement, making it less likely to reach new users.

Furthermore, negativity not only attracts attention but can also repel it. When content provokes fear, moral discomfort, or emotional overload, it may cause users to disengage, limiting the number of resharers, i.e., people willing to reshare it. According to the Extended Parallel Process Model [75], when individuals perceive a threat as emotionally overwhelming and believe they lack the efficacy to respond effectively, they may engage in fear control responses, such as emotional withdrawal or disengagement, to protect themselves. These responses further reduce the susceptible pool of audiences who could reshare.

In sum, while negative emotions may enhance initial resharing, they can also trigger withdrawal, network saturation, and algorithmic suppression, especially in emotionally unambiguous, tragic events. These processes collectively contribute to shorter cascade lifetimes. Hence, we propose:

*Hypothesis 3: Social media messages containing negative-valence emotions are reshared for a shorter period than messages containing neutral or positive-valence emotions.*

### Arousal and resharing speed

Resharing speed refers to how quickly an original message typically propagate across social media once posted. Prior studies confirm that sentiment can accelerate this process. Stieglitz and Dang-Xuan [8] modeled speed as the time lag between an original tweet and its first retweet, showing that emotionally expressive tweets spread faster than neutral ones. Tsugawa and Ohsaki [43] measured speed as the time required for tweets to reach specific cascade sizes (e.g., 10, 100, or 1,000 retweets) and found that negative tweets tended to travel more quickly than positive or neutral ones when diffusion volume was high. Together, these findings highlight that resharing speed captures the collective responsiveness to a message, with emotionally charged content prompting more frequent and synchronized reactions.

High-arousal emotions are especially effective at increasing resharing speed due to multiple reinforcing factors. First, Prior research argues that high arousal enhances action tendencies and mobilizes individuals toward expressive behaviors such as resharing [71]. Such emotions heighten urgency, prompting users to react in closer temporal proximity, which at the collective level produces faster and more synchronized cascades. Empirical evidence supports this logic: Fan et al. [76] show that anger, a high-arousal negative emotion, diffuses more rapidly than joy because it prefers weaker ties, which enables it to penetrate more widely across the network (engaging more audience promptly), and produces shorter retweet intervals, which reduces delays between consecutive reshares. Together, these findings suggest that arousal acts as a catalyst for resharing speed, shaping not only whether content spreads but also how quickly a collective responds.

Second, in the context of celebrity suicides, these dynamics are amplified. Unlike rumors or ongoing news, celebrity suicides are sudden, dramatic, and episodic, producing cascades that are intense but short-lived [25,63]. High-arousal emotions in this setting are expected to increase speed by reinforcing homogeneity of reaction, as grief and shock are present in the context (widely shared) and foster emotional mimicry, leading individuals to align with one another and respond in more simultaneous and concurrent ways [53]. Third, platform algorithms further compound this process by promoting content that gains traction quickly, creating feedback loops where early surges in resharing accelerate subsequent exposure [21]. As a result, high-arousal content triggers broad resonance, which manifests as reshares occurring in closer adjacency to one another, due to the combined effects of individual mobilization, collective synchronization, and algorithmic amplification.

*Hypothesis 4: Social media messages containing high-arousal emotions are reshared more quickly than messages containing neutral or low-arousal emotions.*

### Arousal and resharing burstiness

Burstiness describes a temporal pattern of engagement characterized by short, intense flurries of activity followed by periods of minimal or no engagement. This phenomenon has been observed in various human communication systems, such as email and instant messaging, which points to the uneven micro-level tendencies of users to allocate attention, memory, and action resources [77]. In social media, burstiness reflects the clustering of retweets within narrow time windows, producing spikes in engagement that are not evenly distributed across a cascade’s lifetime.

Building on prior research, we expect high-arousal emotions to be strongly associated with bursty patterns of resharing. As we noted previously, these emotions stimulate rapid mobilization [71], activating intense reactions within emotionally aligned user groups, particularly in the wake of emotionally unambiguous events like celebrity suicides. Additionally, Social media collectives are inherently clustered, with users embedded in tightly knit communities [78]. High-arousal content is more likely to activate each of these emotionally synchronized members within these clusters. Once a cluster is triggered, users’ emotional alignment and social proximity lead to rapid, localized sharing, causing short-lived spikes in activity. Previous studies have shown that highly emotional and negative content creates brief but intense engagement spikes [9,47]. This pattern of rapid intra-cluster saturation, followed by temporary breaks before engagement spreads to other clusters, creates a cascade characterized by multiple bursts rather than sustained, uniform activity.

In contrast, low-arousal emotions are less likely to activate various clusters and spread rapidly across them. Hence, they diffuse more slowly and evenly, resulting in flatter or less pronounced engagement patterns. The emotional salience of high-arousal messages, combined with the topology of clustered networks, thus contributes to temporal irregularity in resharing behavior. Therefore, we propose:

*Hypothesis 5: Social media messages containing high-arousal emotions show more bursty resharing patterns than messages containing neutral or low-arousal emotions.*

Further, while we attempt to extend prior insights to the current empirical context, a contrasting theoretical approach to understanding emotional content is that theoretical frameworks (such as valence-arousal) are relatively less predictive of the virality of emotional content than models that separately examine discrete emotions [28,32]. We will discuss these issues in the implications.

## Empirical analysis

### Data collection

To examine the temporal dynamics and emotional composition of public responses to celebrity suicides, we identified four incidents that occurred between 2012 and 2013 and analyzed the related activity on Twitter (recently renamed X in July 2023). The analysis covered both original tweets and retweets generated in the aftermath of each event. We employed a combination of textual, temporal, and network features derived from the tweets and user activities to characterize communication patterns following the incidents. The selected celebrities, listed in Table 1, represent prominent figures from television, cinema, and sports. For ease of reference, their names are abbreviated as DC, JS, LTY, and TS. The procedure for identifying these cases and collecting the corresponding data is detailed in S1 Appendix.

**Table 1:** Demographics and general characteristics of suicide events.

Name	Occupation	Age	Suicide method	Suicide date	#Tweets (#Users)
Don Cornelius (DC)	TV and Film	76	Gunshot in home	Feb 1, 2012	317,457 (222,698)
Junior Seau (JS)	Sports	43	Gunshot in home	May 2, 2012	406,972 (273,344)
Tony Scott (TS)	Film	68	Jumping from bridge	Aug 20, 2012	204,853 (120,670)
Lee T. Young (LTY)	Film	29	Gunshot in home	Aug 19, 2013	142,621 (93,203)

The dataset comprises all posts shared on Twitter within 21 days following each suicide. This timeframe is consistent with prior research indicating that the population-level influence of celebrity suicides typically diminishes within two to three weeks [64]. The 21-day window thus provides a conservative and standardized observation period, given the daily activity counts in our data. Each entry in the dataset represents either an original tweet or a retweet, totaling approximately 1.1 million posts. In this structure, every tweet constitutes a distinct observation, and each of its retweets is recorded as an additional observation. For example, if tweet A is retweeted three times (rA1, rA2, rA3), the resulting cascade includes four posts: one original and three reshares.

Each post in the dataset includes three sets of variables: content variables (word count, dominant emotion, and words-per-sentence) which reflect the linguistic characteristics of the tweet; temporal variables (the time between the original tweet and last retweet and the time difference between subsequent retweets) pointing to the conversational features of the posts; and structural variables (follower/friend count, exposure, retweet count) exhibit how the network of users is formed around every single post on the platform. A descriptive guide table of the tweet variables is provided in S1 Appendix (S1 Table 2).

### Identifying resharing cascades

Following earlier studies on online social media [1,36,79], we employ the notion of a “cascade” as the series of reshares (retweets) occurring for a single tweet. Therefore, if a tweet is retweeted *n* times, a cascade of size *n + 1* is generated, with its lifetime being the timespan between the last retweet and the original tweet.

Following the four suicide events (DC, LTY, JS, TS), 91,868 distinct cascades were created on Twitter in the time window of 21 days after each suicide date. These were identified by tweets that were retweeted at least once. The distribution of cascade size in terms of the number of retweets is right-skewed, as expected, for online user-generated content. Sixty-seven percent of the cascades consist of one original tweet and one retweet, thus having a size of two, and only 8% involve more than five retweets. We list the summary statistics of cascade variables in Table 2. Author statistics in this table refer to the original author alone and not the retweeters in that cascade.

**Table 2:** Summary statistics of cascade variables.

Variables	Range	Mean	SD
Word Count	3 – 37	16.30	7.21
Hashtag	0,1	0.28	–
Size	2 – 6,111	5.36	40.17
Author Follower Count	0 – 13.5M	23,927	228,165
Author Friends Count	0 – 747K	1,750	8,864
Author Status Count	1 – 654K	18,058	28,923
Author Like Count	0 – 599K	356	4,204
Author Verified	0,1	0.04	–

When users retweet (reshare) a tweet, it becomes visible to the followers of the user who retweeted it. This sets off a chain reaction, allowing a new set of users to read and engage with the content. If a user from this second set decides to retweet, the tweet then becomes visible to their followers, and the process continues. This iterative resharing process forms a cascade graph illustrating the order and relationships among the users involved in the retweeting sequence.

However, while we have the retweet time stamp and the user ID of the retweeter, data on which specific retweet instance was read and reshared by the user is unavailable. For example, if user Y retweets a tweet from user X, and then user Z retweets the retweet from user Y (because Y viewed Z’s retweet as Z’s follower or by any other means), our data records two retweet instances, each representing a pair of the original author and the final retweeter: (X, Y) and (X, Z). Thus, our data does not include information on which intermediate actor (Y), if any, resulted in Z resharing the original tweet (that is, the contagion chain X-Y-Z). Despite this limitation, we can create a time series of retweets within each cascade by recording the temporal footprint of each node in the cascade graph (where each node represents a retweet instance). Thus, although the sequence order cannot capture the exact relationships among users, it remains a valuable approach for studying contagion dynamics. This methodology has been previously employed in research to analyze temporal sequences of human actions and communications, providing insights into group and individual behavior under various conditions, such as excitement and stress [80–82].

### Emotion detection

To quantify the emotional content of a tweet, we extracted emotion scores for each cascade using the DistilRoBERTa model, a type of Bidirectional Encoder Representations from Transformers (BERT) model [34]. Specifically, we use a fine-tuned DistilRoBERTa-base checkpoint, trained on datasets such as Twitter and Reddit, to identify six basic emotions (anger, disgust, fear, joy, sadness, surprise) and neutral content [83]. The performance of this type of language model on natural language processing tasks has been reported to be on par with that of humans and is well-suited for detecting emotions in our dataset [84]. The architecture of this model is similar to BERT, which uses a deep neural network model and self-attention mechanism with compression such that the resulting model is more efficient and smaller. The model can recognize multiple emotions for each tweet and assign probability scores for each associated emotion, with the probability scores adding up to one. Each tweet was then characterized by a dominant emotion identified through the highest probability score. For example, a message identified as having an anger score of 30% and a sadness score of 70% would then be categorized as having the dominant emotion of sadness.

Model validation was performed on a random subset of 1000 tweets from our dataset that was annotated by Prolific users for the basic emotion types (sadness, neutral, surprise, anger, joy, disgust, and fear) to ensure that the model is relatively accurate in identifying the dominant emotion. Using human annotations as ground truth, the DistilRoBERTa model achieved a weighted average for precision, recall, and F1 scores of 0.45, 0.29, and 0.35, respectively. Accuracy scores for individual emotional categories mostly outperform results obtained from weighted random guessing. Details of the validation and the results of the validation are provided in S2 Appendix.

**Fig 1:**
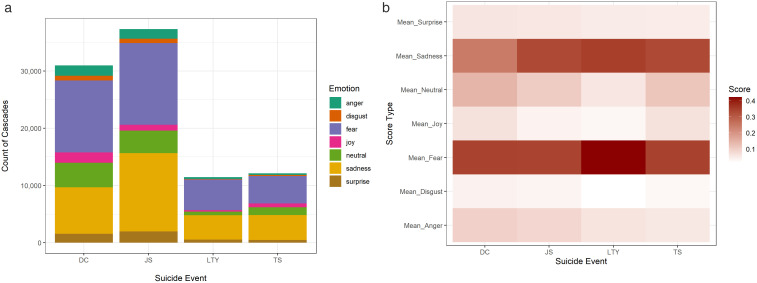
(a, left) Frequency and (b, right) intensity of emotions in the cascades.

Our analysis of the cascades’ dominant emotion indicates that 40% of cascades signal the dominant emotion of fear, followed by 33% of sadness, 11% being neutral, and the remaining 12% indicating either surprise, anger, joy, or disgust as their dominant emotion. Fig 1a displays the composition and frequency of these negative emotions in the cascades of each suicide event. Looking beyond the dominant emotional label, we also analyzed the mean probability score of each emotion within the four suicide events. Our findings on the intensity of emotions (Fig 1b) indicate that fear and sadness are generally more pronounced compared to other emotions and that the intensity of emotions also varies with regard to each event, indicating how users tend to express a different composition of emotions under different conditions. For example, the cascades in LTY and JS events exhibit more emotional content and less neutrality, which can be attributed to their popularity and the public’s general perception. Lee Thompson Young (LTY) is the youngest of these four celebrities. Therefore, the composition of his fan base and the fact that people are more affected by the death of a young person may explain why the intensity of fear and sadness is highest in the aftermath of that event [25].

Table 3 lists the count of cascades labeled with each dominant emotion. This categorization shows that fear and sadness are the most predominant emotions in cascades, followed by surprise, anger, joy, and disgust. S1 Table 3 (S1 Appendix) shows sample tweets from each category with their emotion scores.

**Table 3:** Percentage of cascades in each event labeled with each dominant emotion.

Emotions	DC	JS	TS	LTY	All Events
Anger	5.8	4.5	2.3	2.7	4.4
Fear	40.6	38.3	39.3	47.0	40.3
Sadness	26.2	36.7	35.8	37.2	33.1
Joy	5.8	2.7	5.6	2.0	4.0
Disgust	2.6	2.0	1.7	0.7	2.0
Surprise	5.1	5.3	4.0	4.8	5.0
Neutral	13.8	10.5	11.1	5.5	11.1

### Operationalizing propagation metrics

*Cascade Size:* Consistent with prior work [1], we define cascade size as the number of retweets plus one, capturing all nodes in the cascade, including the original tweet.

*Cascade Lifetime:* Following prior work [27,36], we compute cascade lifetime as the duration between the first and last tweet. It is important to note that while our data contains retweets up to 21 days after the event, it is unlikely that this truncated window would result in us missing any activity (i.e., retweets) related to the event. This is because social media activity around shocking events, including on Twitter, drops to negligible levels after 2–3 weeks [63]. As summarized in Table 4, cascade lifetimes in our dataset range from 0 to 28,600 minutes (roughly 20 days), with a mean of 218 minutes (3.6 hours).

**Table 4:** Summary of propagation measures: structural and temporal.

Celebrity	Size		Lifetime *(Minutes)*		Median Delay *(Seconds)*		Time-to-fifth RT (Seconds)		Burstiness (σ/μ)	
	mean	sd	mean	sd	mean	sd	mean	sd	mean	sd
DC	5.02	40.46	191	1.1K	4,141	31K	5,833	38K	1.35	1.33
JS	5.06	42.48	128	636	3,249	21K	3,255	15K	1.35	1.33
TS	6.41	33.61	412	1.5K	8,203	38K	9,487	49K	1.58	1.47
LTY	6.17	37.97	376	1.6K	5,802	39K	9,907	47K	1.52	1.31
ALL	5.36	40.14	218	1.1K	4,522	30K	6,282	36K	1.40	1.35

*Time to Nth ReTweet:* Based on existing measures [8,47], we first identify tweets that received at least five retweets (8,812 cascades) and then calculate the time taken to reach the fifth retweet; The time-to-fifth retweet ranges between 7 seconds to over 14 days, distinguishing between influential tweets that either gained traction rapidly or over an extended period.

*Median Delay*: We propose *median delay* as another measure of retransmission speed, defined as the median amount of time elapsed between each two consecutive retweets within a cascade. This measure has not been used in prior studies, but the median delay can reveal another beneficial aspect of the temporal dynamics of a cascade. A cascade with a slight median delay indicates a fast sequence of resharing, which may happen because of the social network structure in which the authors and retweeters are a part or because of the rapid influence it has on the audience who reshare (adopt) the content, or both. Either way, median delay, being less biased than the average delay, can appropriately capture the dynamics of tweet retransmission. Our measure improves upon the previous measure of time-to-Nth retweets since, by definition, we may be excluding many tweets from the analysis. The summary statistics in Table 4 show that the median delay, as defined in our data, can range from zero (usually smaller cascades that receive rapid reshares) to over a million seconds (again, small cascades that took a long time to get reshared). On average, cascades in the JS suicide event demonstrated smaller ranges of median delay, showing that content related to this event was reshared faster compared to the others.

*Burstiness:* In our context, this can manifest as a (temporal) sequence of retweets within each cascade, where subsets of retweets are posted rapidly within short intervals of time. Intuitively, the greater the intensity of an emotion, or if the emotions fall within a specific category of valence or arousal [31], the more likely reactions to them will be clustered in time. To assess the degree of burstiness of a cascade, we employ the coefficient of variation (CV), calculated as the ratio of the standard deviation to the mean of inter-retweet delays (σ/μ) as the most straightforward measure of irregularity [85]. A CV value exceeding one implies that events are more clustered or irregularly spaced than a random process. In contrast, a CV value below one indicates a more uniform spacing of events.

**Fig 2:**
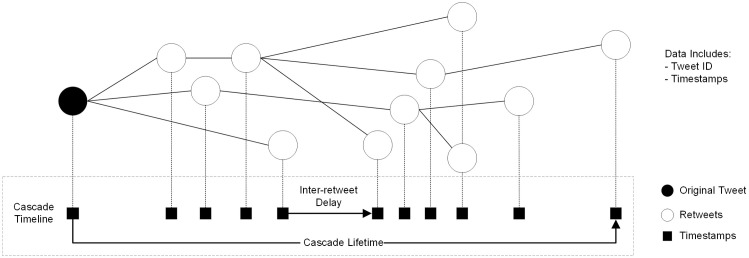
Cascades’ temporal structure and data generation process.

The characteristics of the retweet cascade data and the details of the outcome variables are represented in Fig 2. Table 4 presents the descriptive statistics for all the above propagation outcomes. We use these five measures to run a series of analyses, which are presented in Table 5 (Size and Log Lifetime), Table 6 (Median Delay and Time-to-fifth RT), and Table 7 (Burstiness).

**Table 5:** Regression results on the lifetime and size of cascades.

	DV: Cascade Size *Negative Binomial Model*				DV: Log (Lifetime) *OLS Model*			
	Size ≥ 2		Size ≥ 3		Size ≥ 2		Size ≥ 3	
	(1)	(2)	(3)	(4)	(5)	(6)	(7)	(8)
Emotion: Anger		−0.057^***^		−0.042		−0.155^***^		−0.200^***^
Emotion: Fear		−0.099^***^		−0.103^***^		−0.075^***^		−0.100^**^
Emotion: Sadness		−0.081^***^		−0.076^***^		−0.003		−0.052
Emotion: Joy		−0.087^***^		−0.095^***^		0.190^***^		0.252^***^
Emotion: Disgust		0.622		0.858^**^		0.215^*^		0.208^***^
Emotion: Surprise		−0.038		0.007		−0.174^***^		−0.223^***^
# Auth. Followers (Log)	0.332^***^	0.336^***^	0.317^***^	0.325^***^	0.429^***^	0.427^***^	0.399^***^	0.398^***^
# Auth. Friends (log)	−0.110^***^	−0.109^***^	−0.094^***^	−0.092^***^	−0.157^***^	−0.157^***^	−0.125^***^	−0.125^***^
# Auth. Tweets (log)	−0.123^***^	−0.125^***^	−0.126^***^	−0.130^***^	−0.343^***^	−0.339^***^	−0.366^***^	−0.361^***^
# Auth. Likes (log)	0.037^***^	0.033^***^	0.043^***^	0.036^***^	0.048^***^	0.047^***^	0.062^***^	0.061^***^
Auth. Verified?	0.820^***^	0.818^***^	0.585^***^	0.571^***^	0.887^***^	0.886^***^	0.507^***^	0.505^***^
Wordcount	0.011^***^	0.009^***^	0.018^***^	0.014^***^	0.012^**^	0.012^**^	0.021^**^	0.020^**^
Hashtag?	−0.005	0.046	−0.028	0.044	0.127^***^	0.162^***^	0.072	0.113^**^
Celebrity: JS	−0.061^***^	−0.039^**^	−0.116^***^	−0.060^**^	0.045^**^	0.048^***^	−0.004	0.009
Celebrity: LTY	−0.017	0.024^***^	−0.043^**^	0.036^***^	0.366^***^	0.371^***^	0.627^***^	0.643^***^
Celebrity: TS	−0.278^***^	−0.243^***^	−0.369^***^	−0.297^***^	0.547^***^	0.548^***^	0.501^***^	0.512^***^
Intercept	0.437^**^	0.488^***^	0.806^***^	0.832^***^	3.178^***^	3.186^***^	4.048^***^	4.069^***^
Observations	91,868	91,868	30,637	30,637	91,868	91,868	30,637	30,637
Adjusted R^2^	–	–	–	–	0.162	0.164	0.244	0.246
Log Likelihood	−207,590	−206,767	−92,816	−92,346	–	–	–	–
Akaike Inf. Crit	415,202	413,569	185,655	184,726	–	–	–	–

Note: *p < 0.1; **p < 0.05; ***p < 0.01; The standard errors in this linear regression model are clustered on the celebrity suicide event; hence, four clusters are created. Negative Binomial regression results are generated using the MASS library in R.

**Table 6:** Regression results on the median delay and time-to-fifth RT of cascades.

	DV: Log (Median Delay) *OLS Model*		DV: Log (Time-to-Fifth RT) *OLS Model*	
	Size ≥ 6		Size ≥ 6	
	(1)	(2)	(3)	(4)
Emotion: Anger		−0.139^**^		−0.227^***^
Emotion: Fear		0.100		0.210^**^
Emotion: Sadness		0.057		0.102
Emotion: Joy		0.351^***^		0.308^***^
Emotion: Disgust		−0.301^***^		−0.411^***^
Emotion: Surprise		−0.184^**^		−0.162
# Auth. Followers (Log)	−0.079	−0.086^**^	−0.249^***^	−0.259^***^
# Auth. Friends (log)	−0.009	−0.009^*^	0.019^***^	0.019^***^
# Auth. Tweets (log)	−0.116^***^	−0.112^***^	−0.051	−0.049
# Auth. Likes (log)	−0.015^***^	−0.014^***^	−0.035^***^	−0.033^***^
Auth. Verified?	−0.228^***^	−0.219^***^	−0.394^***^	−0.379^***^
Wordcount	0.009^*^	0.009^*^	0.004	0.005
Hashtag?	0.002	−0.048	0.023	−0.081
Celebrity: JS	−0.039	−0.050	−0.105^***^	−0.130^***^
Celebrity: LTY	0.439^***^	0.414^***^	0.259^***^	0.214^***^
Celebrity: TS	0.696^***^	0.674^***^	0.747^***^	0.707^***^
Intercept	6.552^***^	6.538^***^	9.259^***^	9.246^***^
Observations	8,812	8,812	8,812	8,812
Adjusted R^2^	0.108	0.114	0.157	0.162

Note: *p < 0.1; **p < 0.05; ***p < 0.01; The standard errors in this linear regression model are clustered on the celebrity suicide event, hence four clusters are created.

**Table 7:** Regression results on the burstiness of cascades (coefficient of variation).

	DV: Coefficient of variation *OLS Model*	
	(1)	(2)
Emotion: Anger		0.117^***^
Emotion: Fear		−0.226^***^
Emotion: Sadness		−0.083
Emotion: Joy		−0.206^***^
Emotion: Disgust		0.609^**^
Emotion: Surprise		0.055
# Auth. Followers (Log)	0.340^***^	0.350^***^
# Auth. Friends (log)	−0.074^***^	−0.074^***^
# Auth. Tweets (log)	−0.188^***^	−0.188^***^
# Auth. Likes (log)	0.058^***^	0.056^***^
Auth. Verified?	0.355^***^	0.338^***^
Wordcount	0.014^***^	0.013^***^
Hashtag?	−0.007	0.114
Celebrity: JS	−0.098^***^	−0.066^*^
Celebrity: LTY	0.106^***^	0.154^***^
Celebrity: TS	−0.322^***^	−0.277^***^
Intercept	0.947^***^	0.939^***^
Observations	8,812	8,812
Adjusted R^2^	0.172	0.177

Note: *p < 0.1; **p < 0.05; ***p < 0.01; The standard errors in this linear regression model are clustered on the celebrity suicide event, hence four clusters are created.

### Regression model

The common model specification for all these regressions is as follows. Each regression model examines the propagation measure as the dependent variable (DV) (at the cascade level, log-transformed where necessary to account for the distribution’s skewness), as a function of several independent variables (Table 2), which include the tweet’s emotional content, linguistic features, and the original author characteristics (popularity and activity). The transformations of specific propagation measures yielded an approximately normal distribution of the variables. Since the emotion of a tweet is defined only by the dominant emotion, for each cascade, only one of the six dummy independent variables is coded as ‘1’, else ‘0’. Thus, estimation is conducted with Neutral as the base emotion (See Table 3). The controls in the model include friends count, status count, favorites count, word count, hashtags, and fixed effects for each celebrity (Eq 1).


$$\begin{array}{cc} D.V.= & \beta_{\text{0}}+\beta_{\text{1}a}Anger+\beta_{\text{1}b}Fear+\beta_{\text{1}c}Sadness+\beta_{\text{1}d}Joy+\beta_{\text{1}e}Surprise\\ & +\beta_{\text{1}f}Disgust+\beta_{\text{2}}Celebrity+\beta_{\text{3}}CONTROLS+\epsilon_{i} \end{array}$$ (Eq 1)

Since each observation represents a distinct cascade resulting from a specific tweet, the coefficient estimates $\left(\beta_{\text{1}a}-\beta_{\text{1}f}\right)$ from the above regression will indicate how the emotional content within each tweet (the independent variables) is correlated with the various propagation metrics (the dependent variable). The results will thus indicate the role of various emotions contained within tweets in triggering resharing in distinct ways, depending on which metric(s) it strongly correlates with.

## Results

### Cascade size

The average cascade size is 5.36 (SD = 40.2), indicating substantial variation in diffusion patterns. To address the over-dispersed nature of the count data, we employed a negative binomial regression model [86] and, for comparison, fitted a Poisson model to the independent variables. As cascades expand and reach wider audiences, the likelihood of additional reshares increases, suggesting that the dynamics of large cascades differ meaningfully from those of small ones. Accordingly, we analyzed two subsets of data: (1) the full sample of 91,868 cascades, where a single retweet corresponds to a cascade size of 2 (including the original tweet), and (2) cascades with three or more posts (size ≥ 3), totaling 30,637 observations. The regression results for cascade size across these subsets are reported in Table 5. We acknowledge that sampling on the dependent variable may limit generalizability. The size threshold was set to improve construct validity by focusing on cascades that show meaningful network diffusion beyond isolated or incidental reshares. This approach helps reduce noise from the large number of trivial cascades that dominate the distribution.

Emotions demonstrate a variety of effects on cascade size (Table 5, Models 2 & 4). Prior studies have typically examined emotions as just positive and negative, but we note that there are nuances amongst negative emotions. Except for surprise and disgust, the emotions of fear, sadness, anger, and joy reduce the size of cascades compared to the baseline of neutral emotion. Disgust has a positive effect on size in Model 4. Among the control variables, word count positively influences cascade size, and hashtags do not have any significant effect. The dummy variables for celebrity indicate significant effects on cascade size (LTY > DC > JS > TS). The popularity of original authors (# Auth. Followers) has a positive effect on size, whereas their friendliness (i.e., the number of people they follow: # Auth. Friends) decreases cascade size. The verified status of the authors on Twitter (Auth. Verified) positively influences cascade size. Finally, the activity level of the original authors, including the total number of tweets they posted in their account’s lifetime (# Auth. Tweets), is negatively related to cascade size. In contrast, the total number of tweets they liked (# Auth. Likes) increases the cascade size. Interestingly, not only emotions but also the author’s characteristics, including their follower count and activity levels, affect the virality of their content distinctly.

### Cascade lifetime

The size of the cascades is intuitively correlated with their lifetime (r=0.333,p=0.000), suggesting that content that persists longer usually receives more retweets, too. There are two overall observations about the coefficients. First, similar to cascade size, there is a significant contextual difference between the four suicide events, indicating that the celebrity in question influences the longevity of cascades. Second, with some exceptions, the coefficients for control variables are remarkably similar in size and significance across the two metrics of Cascade Size and Lifetime – but not the coefficients for the emotion categories. Emotions exhibit a diverse and nuanced set of effects. The cascades where the dominant emotion is either anger, fear, or surprise lead to a reduced lifetime compared to those containing disgust and the positive emotion of joy, which extend the cascade.

The coefficients for control variables are similar to those observed for Cascade size except for Hashtag, which is significant here (Table 5, Models 6 & 8). Thus, hashtags have a positive effect on the lifetime of the cascades, suggesting that algorithmic hashtag-based recommendations can contribute to the diffusion by extending the cascades’ lifetime [45] by maintaining the visibility of the tweets for a longer period of time. Furthermore, more prominent authors, i.e., those with a larger pool of followers (# Auth. Followers), generate cascades with longer lifetimes (Model 6, β=0.427,p=0.000,CI=0.030) whereas a greater number of friends of the tweeted author is associated with a reduced lifetime (Model 6, β=−0.157,p=0.000,CI=0.017).

### Median delay

Next, as a measure of cascade speed [82], we examined the median inter-retweet delay in the temporal sequence by which cascades were reshared. A lower median delay represents a high cadence of retweets within each cascade. Larger delays in retweeting suggest lower intensity with which the tweet corners attention from the users, a lower cadence. We drop all tweets with less than five retweets for our analysis to focus on highly contagious cascades with rich information in their resharing pattern. As before, this leaves us with 8,812 observations (cascades). The results of the analysis are presented in Table 6 (Models 1 & 2).

Our results suggest that the emotions of joy and fear make tweets get retweeted slower by increasing the median delay, as seen in the models above (Table 6, Model 2). The resulting sequence of Twitter activity would be relatively dispersed, with separate incidents of events (retweets) taking place at longer intervals of time. Meanwhile, the emotions of anger (Model 2, β = −0.139, p = 0.019, CI = 0.059), disgust (Model 2, β = −0.301, p = 0.00, CI = 0.069), and surprise (Model 2, β = −0.184, p = 0.00, CI = 0.079) increase the resharing speed, controlling for all contextual, linguistic, and network-related factors.

We also observe that the authors’ activity level positively influences the speed of future reshares. Furthermore, popular users with higher follower count create cascades with faster resharing rates (Model 2, β = −0.086, p = 0.045, CI = 0.043). As expected, this fast and long-lasting effect makes emotions and ideas viral and more potent in influencing additional users. Unlike cascade lifetime, here the effect of author friendliness (number of users they follow, # Auth. Friends) is also slightly positive towards cascade speed by decreasing the median delay (Model 2, β = −0.009, p = 0.068, CI = 0.005), which means that although they create shorter living cascades, their content can still generate slightly faster responses by the audience.

### Time to 5^th^ Retweet (Time5RT)

Like the median delay, this measure also accounts for the speed at which cascades grow. The effects of anger and disgust remain positive and significant, but joy has a negative effect. The results (Table 6, Model 4) also show that fear, which used to be ineffective towards median delay, can increase Time5RT (Model 4, β = 0.210, p = 0.016, CI = 0.086) and thus slow the resharing speed. Furthermore, the effects of author characteristics (popularity and activity) are similar to the effects they have on median delay, except for author activity (# Auth Tweets), which does not show any significant effect on Time5RT. The nuanced difference across the coefficients for these two measures (median-delay vis-a-vis Time5RT) could be clarified by the non-uniform distribution of the temporal process through which these retweets occur, such as bursts of activity in brief time spans.

### Cascade burstiness

Here, the results (Table 7, Model 2) show that the emotions of anger (Model 2, β = 0.117, p = 0.004, CI = 0.04) and disgust (Model 2, β = 0.609, p = 0.031, CI = 0.282) generate bursty and clustered temporal sequences of retweets: whereas tweets containing fear (Model 2, β = −0.226, p = 0.003, CI = 0.075), and joy (Model 2, β = −0.206, p = 0.000, CI = 0.031) generate less bursty patterns.

## Discussion of results and limitations

Our findings show that emotions do play a role in resharing, but their effects are not uniform across valence-arousal categories [28]. This partly reflects our empirical strategy: while the hypotheses were framed around the bipolar dimensions of arousal and valence (four categories), our analysis moved beyond this structure by examining six distinct emotions identified through a state-of-the-art large language model (DistilRoBERTa) [34]. This more granular lens reveals that emotions grouped within the same quadrant of the circumplex model (e.g., high-arousal–negative valence) [31] do not always exhibit consistent associations with propagation. In this sense, the hypotheses serve as a theory-driven starting point, while the empirical results highlight the complexity of how specific emotions shape propagation.

The results of our hypothesis testing, based on six distinct emotions, are presented in Table 8. In the context of celebrity suicides, which are highly tragic events with global resonance, our data show that social media reactions are marked by extreme responses, most often characterized by negative valence and high arousal. Given the broad appeal of celebrities, these dynamics expose a wide audience to heightened negative emotions through online discourse.

**Table 8:** Summary of results.

1	2	3	4	5	6	7
Emotion^*^	Size	Lifetime	Speed	Burstiness	Valence	Arousal
Anger	(-)^**^	(-)	(+)	(+)	Negative	High
Disgust	(+)	(+)	(+)	(+)	Negative	High
Surprise	(0)	(-)	(+)	(0)	Neutral	High
Sadness	(-)	(0)	(0)	(0)	Negative	Low
Fear	(-)	(-)	(-)	(-)	Negative	High
Joy	(-)	(+)	(-)	(-)	Positive	Low

* The effect of emotions on cascade metrics is presented compared to neutral content.

**(+)/(-)/(0) indicates a positive/negative/neutral effect on the outcome variable.

Hypothesis 1 proposed that high-arousal messages would have larger cascade sizes than neutral messages; This was supported for disgust but contradicted for anger and fear, which had smaller cascade sizes than neutral tweets. Hypothesis 2 proposed that high-arousal messages would have shorter lifetimes, a counterintuitive expectation based on prior research conducted in a different empirical context. This hypothesis received stronger support, as anger, fear, and surprise were associated with shorter lifetimes, although disgust showed a longer lifetime. Additionally, joy exhibited a longer lifetime compared to neutral tweets. Hypothesis 3 proposed that negative-valence tweets would have shorter lifetimes than neutral and positive-valence tweets. The results were mixed: anger and fear had shorter lifetimes, while disgust again exhibited a longer lifetime. Hypothesis 4 proposed that high-arousal messages would spread faster. Here, this was supported for anger and disgust, both of which propagated more quickly, whereas fear propagated more slowly than neutral tweets. Finally, hypothesis 5 proposed that high-arousal messages would exhibit greater burstiness. This was partially supported, as anger and disgust showed greater burstiness, while fear showed the opposite pattern.

Beyond the formal hypothesis tests, several additional patterns emerge. Sadness does not differ substantially from neutral tweets in any propagation metric. Disgust stands out as a dominant emotion, positively influencing cascade size, lifetime, speed, and burstiness, suggesting that tweets expressing disgust propagate more extensively, persistently, and intensely in the aftermath of celebrity suicides. In contrast, fear exhibits a negative effect across all metrics, indicating a highly subdued propagation pattern. Although both disgust and fear are high-arousal negative emotions, they display opposite propagation characteristics. Among other high-arousal emotions, surprise is notable for showing divergent effects on size and lifetime, counter to the intuition that larger cascades should statistically correlate with longer lifetimes. Finally, while joy results in smaller cascade sizes, it is associated with longer lifetimes compared to neutral and negative content.

Our study has several limitations. First, we analyzed only tweets that triggered cascades, so our conclusions are generalizable only to messages that were reshared. A more comprehensive analysis would also model the probability of a message being reshared in the first place. Second, features and algorithms on social media platforms, as well as the composition of their user bases, may evolve over time, potentially affecting propagation mechanisms and further limiting the generalizability of our findings. Third, our analysis relies solely on the timestamps of retweets, without capturing the precise sequence of how each cascade unfolded (Fig 2). Future research could incorporate the structural connections among users within a cascade, if such data become available, to better understand how interventions might interrupt the spread of harmful content across different network structures.

Additionally, our study does not directly test the assumption that emotions expressed in content are also experienced by users and shape their resharing behavior. Addressing this would require experimental or survey-based methods that capture user-level emotional responses. We therefore distinguish between resharing behavior, which drives propagation and is the focus of our analysis, and emotional contagion, which entails inducing the same emotion in the audience (See S1 Appendix for conceptual clarification). While the broader motivation for studying propagation is to understand mechanisms that may contribute to self-harm, our evidence speaks only to a potential mediating pathway rather than behavioral contagion itself. Future research could extend this work by linking social media data with population-level suicide statistics [64] to examine these broader outcomes.

## Implications

Several insights emerge from our findings that have implications for research on the propagation of emotional content. First, our results reinforce the growing consensus that theories of emotional propagation must move beyond coarse classifications by valence [9,15,43] and arousal [33]. Recent work [28,32] has argued that discrete emotions provide a more accurate lens for understanding diffusion dynamics, and our study adds further empirical support. We add that emotions within the same quadrant of the circumplex model of affect do not propagate in uniform ways, particularly accounting for their temporal patterns. Anger, fear, sadness, and disgust are all negatively valenced, yet they differ in how quickly they spread, how long they persist, and the shapes of their cascades. Some emotions spread rapidly but fade quickly, while others persist longer despite slower uptake. These findings underscore the importance of examining emotions at a more granular level, where their unique appraisals, action tendencies, and social meanings shape how they diffuse through networks. By documenting such heterogeneity within negative emotions, our study provides additional evidence that discrete emotions are better suited than broad valence–arousal categories for capturing the dynamics of emotional propagation.

Second, the findings highlight the critical role of context in shaping how emotions propagate [87]. Theories of content diffusion often treat emotions as universal drivers of spread, but the same emotion can behave differently depending on the event that triggers it. Anger associated with rumors or political conflict may spread rapidly as a mobilizing force [27], while anger in response to a celebrity suicide may be tempered by grief, stigma, or collective mourning. This demonstrates that propagation is not a uniform process but one conditioned by the meaning of the event and the interpretive frames through which audiences engage with it. Theories of emotional propagation must therefore account for context as a moderating factor, recognizing that propagation signatures emerge from the interaction between the type of emotion and the circumstances of its expression [87]. Without such contextual grounding, findings from rumors [27] or natural disasters [11] cannot be meaningfully generalized to the domain of suicides or other health-related crises.

Third, our multidimensional approach demonstrates that propagation cannot be captured by a single metric. Speed, virality, lifetime, and burstiness each reflect different aspects of diffusion, and conflating them obscures important mechanisms. For example, high-arousal emotions may trigger rapid spread (speed), but this can shorten content lifetime as attention is quickly exhausted. Burstiness reflects synchronized imitation, which may be heightened by credibility cues such as verified status. This implies that theoretical accounts of emotional content propagation should explicitly distinguish among these diffusion dimensions and model how they interact. Instead of assuming that more arousal equals more virality, theory should specify which aspect of diffusion—reach, persistence, intensity, or clustering—is most affected. By linking distinct emotions to distinct diffusion signatures, our study provides a foundation for a richer theoretical framework that aligns mechanisms with measurable outcomes.

Fourth, our results imply that propagation dynamics are co-determined by structural and source-level attributes, not only by the emotional qualities of content. Contrary to prior evidence [8,88], hashtags do not significantly enlarge cascades, which suggests that topical labels add little incremental visibility in this context, possibly because attention is already concentrated or because platform ranking treats hashtags as weak relevance signals. In contrast, user popularity and activity (follower counts, posting frequency, accumulated likes) reliably enhance spread, consistent with mechanisms of baseline audience size, repeated exposure, and preferential visibility in feeds. Verified status further accelerates and prolongs propagation, indicating that recognized credibility functions as a signal that lowers audience skepticism, increases perceived importance, and sustains attention over time. Theoretically, models of emotional propagation should therefore integrate source reputation, social capital, and platform affordances as moderators of distinct diffusion dimensions (speed, virality, lifetime, burstiness). These moderators are likely to interact with emotion categories, producing characteristic metric profiles; for example, anger from high-status accounts may convert rapid bursts into longer lifetimes, whereas the same emotion from low-status accounts may dissipate quickly. This shifts explanation from content-only accounts toward joint content–actor–structure models and reframes the null effect of hashtags as a boundary condition on topical signaling rather than a contradiction of prior work.

Beyond these theoretical contributions, the study also has practical implications for communication, public health, and crisis management. Understanding how different emotions propagate can inform more targeted intervention strategies. For instance, anger tends to spread rapidly and in a concentrated burst, suggesting that suicide prevention organizations may need to intervene early when such content appears online. Sadness and fear, while slower to spread or less viral, still expose vulnerable populations to prolonged negativity and may require sustained monitoring. Positive emotions, although less frequent, tend to persist longer and can offer opportunities to amplify resilience-focused messaging. Moreover, the finding that verified users and highly active accounts amplify emotional spread underscores the role of influential voices in shaping online discourse. These insights extend beyond suicide prevention to other domains such as marketing and reputation management, where anger and disgust can quickly damage brand reputation if left unchecked. Overall, the results emphasize the need for fine-grained, emotion-specific approaches to both theoretical modeling and practical intervention [89] in online environments.

## Contribution

While it is well established that social media can facilitate behavioral contagion and that exposure to negative or suicidal content can induce self-harm [12,61,90,91], the role of emotional propagation as a potential mechanism in propagating these adverse effects remains underexplored. Celebrity suicides provide a critical context, as they are associated with documented increases in suicide rates through copycat behavior [22–24,29,57,92,93] and generating deeply negative online discourse [25,62,63]. Existing work has examined the influence of traditional media reports on suicide contagion [24,29,57,94], and more recently, shifts in emotional tone on social media following celebrity suicides [25,62,63], but little is known about how resharing shapes the spreadin patterns of emotions in ways that may exacerbate fatal outcomes. Our study addresses this gap by analyzing multiple propagation metrics to capture the distinct dynamics of different emotions, thereby contributing to the literature on social media–enabled health behaviors and offering a deeper understanding of how emotional propagation may potentially lead to high-risk emotional states, copycat suicides, and the need for tailored intervention strategies.

Our results provide several contributions to the literature on emotional propagation in social media contexts. First, we show that even a tragic event such as a celebrity suicide evokes a spectrum of emotions across both valence and arousal (Table 3). This context provides a unique opportunity to examine how distinct emotions propagate within the same empirical setting, rather than inferring differences from separate events or platforms. We demonstrate that even emotions within the same quadrant of the circumplex model can propagate differently. For example, anger, fear, sadness, and disgust are all negative, yet each follows its own distinct trajectory of spread. By documenting this heterogeneity, our study contributes new evidence on how discrete emotions unfold in response to highly visible tragedies, showing how discrete emotions surpass prior valence–arousal models and extending prior work on settings such as rumors [27,95] and natural disasters [11].

Second, our findings challenge dominant assumptions about the strength of high-arousal negative emotions in driving diffusion [33,71]. Much of the existing literature has suggested that such emotions are the most contagious online, but our results show that this generalization does not hold when propagation is examined across multiple metrics. We analyze four dimensions of propagation: the speed of message diffusion [8,96], the number of users who reshare a message (virality) [8,36,67], its duration in the public sphere (lifetime) [36], and the temporal clustering of reactions (burstiness, reflecting collective mimicry). Anger, for instance, spreads quickly but dissipates rapidly, while sadness circulates widely without sustaining large cascades. Joy spreads more slowly but persists longer, reflecting a different form of endurance. Longer lifetimes indicate content that continues to circulate, showing propagation strength, while faster and more clustered reactions signal highly engaged and homogeneous communities. Taken together, these results indicate that emotional spread and virality should be approached from a broader lens rather than reduced to a single metric. This multidimensional perspective clarifies why past findings appear inconsistent and opens opportunities to develop more complex measures and investigate relationships among dimensions of propagation that remain poorly understood.

Finally, we contribute methodologically by employing advanced computational tools to analyze emotion dynamics at scale. Departing from lexicon-based approaches that dominated earlier work [63], we use a fine-tuned BERT-based language model [34] that achieves higher accuracy in detecting discrete emotions in online text (see S1 Table 3 in the S1 Appendix for validation). This methodological advancement enables us to capture propagation signatures of emotions with greater precision and reliability, strengthening both the empirical foundation and the theoretical claims of our study. Taken together, these contributions enrich understanding of how emotional content spreads in the aftermath of celebrity suicides, situating this high-risk context within broader theories of emotional propagation and extending the methodological toolkit available to future research.

## Conclusion

The advent of online social media is unparalleled in that it is a medium by which individuals’ thoughts, emotions, and opinions are exposed to the world in real time and on a global scale. A social media platform is a venue where social theory is enacted on a global scale. Therefore, analyzing its content offers important insights into human society, especially because it affects the behavior and opinions of those exposed to the media. Of relevance to the current research is how messages that carry emotional content spread through this global platform and reach others by being reshared through a chain of resharing actions by individual users. The underlying premise is that content diffusion differs depending on its emotional content. By analyzing a large dataset of Twitter posts (currently known as X) following tragic and unexpected events, i.e., four celebrity suicides, we uncover interesting patterns of content diffusion and link those patterns to the emotion within the posts.

Emotional dynamics on social media are complex, as emotions embedded in online content can propagate and spread through resharing across large user populations [10,21]. In the case of celebrity suicides, such propagation is especially consequential because it may contribute to behavioral contagion, most notably the “Werther Effect,” where exposure to suicide-related content is associated with increased risk of copycat suicides [24,26]. Prior research has established that celebrity suicides correlate with rises in suicide rates, and the rapid scale and speed of resharing on social media suggest that these risks may be amplified in digital environments [23,25,63]. Our study contributes to this body of work by examining emotional propagation in the aftermath of celebrity suicides, offering evidence on a potential mediating mechanism through which exposure to emotional content may exacerbate vulnerability in audiences. These insights provide a foundation for stakeholders, including public health policymakers, to design more targeted mitigation and prediction strategies in the wake of shocking and tragic events.

## Supporting information

S1 Appendix Conceptual clarification and data description. (DOCX)

S2 Appendix Validation of emotion labels. (DOCX)
