# Supplementary material for: Online propagation of emotions: A study of resharing dynamics on social media following celebrity suicides
Source: PLoS One. 2025 Dec 10;20(12):e0336134. doi: 10.1371/journal.pone.0336134 (PMC12694876; doi:10.1371/journal.pone.0336134)
Supplement: S1 Appendix — (DOCX) [file pone.0336134.s001.docx]

**S1 APPENDIX.**

**Conceptual Clarification and Data Description**

**Conceptual Clarification: Propagation, Diffusion, and Contagion**

In this study, we use the terms *propagation* and *diffusion* interchangeably to denote the observable spread of content across social media networks. We align with a large body of prior research that conceptualizes content diffusion in measurable terms such as cascade size and reshare counts, while distinguishing it from contagion, which involves the transfer of a latent psychological state or behavior to recipients. Although the two processes may co-occur, propagation and diffusion are broader constructs that can happen through resharing, but do not necessarily require contagion (e.g., adoption of the emotion) to take place.

**Data Collection, Variables, and Samples**

Twitter (recently renamed *X* in July 2023) is a major social networking platform with 237.8 million monetizable daily active users (mDAU) as of July 2022 (Twitter, 2022). The data used in this study were collected between 2015 and 2016. We first assembled a list of sports, music, and film celebrities prominent in English-speaking regions (United States and Canada) who had died by suicide. Cases with ambiguous causes of death (for example, Philip Seymour Hoffman, whose death was ruled accidental despite public perception of suicide) were excluded. This process yielded an initial sample of 12 celebrities who passed away between 2009 and 2014.

For each celebrity, research assistants systematically browsed Twitter to identify all hashtags associated with the event. Using a snowball procedure, they expanded these lists by exploring posts containing known hashtags and recording any additional related tags found within those posts. These hashtags were then used to query the complete Twitter data corpus through the Gnip API, which at the time served as the exclusive provider of the full Twitter archive (as opposed to random samples). The query returned counts of tweets and retweets containing the relevant hashtags, which were used to estimate the cost of acquiring the full datasets for each celebrity.

Two cases, Robin Williams (died August 2014) and Amy Winehouse (died July 2011), were removed due to extremely high tweet volumes, which placed the acquisition cost beyond the project’s budget. Finally, we applied two additional selection criteria to refine the sample. First, we prioritized events that occurred within a relatively narrow timeframe to minimize the confounding effects of Twitter’s rapid evolution (e.g., adoption and functionality). Second, we favored more recent cases, closer to 2015, to ensure data relevance. These criteria yielded a final set of four celebrities whose deaths occurred between February 2012 and August 2013.

Using the identified hashtags, we extracted tweets and retweets associated with each of these four events. The specific search queries and corresponding hashtags are listed in S1 Table 1.

S1 Table 1 - Twitter search queries employed for data extraction

| **Celebrity** |  | **Twitter search query strings** |
| --- | --- | --- |
| Don Cornelius |  | ((contains:"don cornelius" OR contains:doncornelius OR contains:"remembering don" OR contains:ripdon OR contains:"tribute to don cornelius" OR contains:"donald cortez cornelius" OR contains:DonaldCortezCornelius) (lang:en)) |
| Junior Seau |  | ((contains:"juniorseau" OR contains: "junior seau" OR contains:"seaufoundation" OR contains:"seau foundation")) |
| Tony Scott |  | (((contains:"vincent thomas bridge" OR contains:"san pedro" OR contains:"True Romance" OR contains:"Beverly Hills Cop" OR contains:"unstoppable" OR contains:"the hunger" OR contains:"spy game") (contains:director OR contains:tony -"tony escalante")) OR (contains:"@tonyscott" OR contains:"tony Scott" OR contains:riptonyscott OR contains:rememberingtonyscott OR contains:"remembering tony scott" OR contains:"rip tony scott")) |
| Lee Thompson Young |  | (((contains:"lee thompson young" OR contains:"lee_T_young" OR contains:ripleethompsonyoung OR contains:leethompsonyoung) OR ((contains:"famous actor" OR contains:"famous star" OR contains:"killed himself" OR contains"so sad" contains:suicide ) (contains:rizzoli OR contains:"jett jackson"))) OR (contains: “age 29” contains:”suicide”) (lang:en)) |

*S1 Table 2 – Description of Cascade Variables*

| Variable | Definition |
| --- | --- |
| Word Count | Count of words in the tweet text (numerical) |
| Hashtag | The existence of hashtags in the tweet text (binary) |
| Dominant Emotion Label (Emotion) | The emotion with the largest probability score (identified by the fine-tuned DistilRoBERTa-based language model) |
| Author Follower Count | Number of users following the original author of the tweet |
| Author Friends Count | Number of users followed by the original author of the tweet |
| Author Status Count | Total number of tweets (posts) written by the original author |
| Author Like Count | Total number of tweets (posts) liked by the original author |
| Author Verified | The verified status of the original author’s account (binary) |
| Size | Total number of tweets in a cascade (original tweet and its retweets) |
| Lifetime | The time distance between the original tweet and its latest retweet |
| Median Delay | Median amount of inter-retweet delay between retweets of a cascade |
| Time-to-fifth RT | The time distance between the original tweet and its fifth retweet |
| Burstiness | The extent to which retweets are clustered in time |

*S1 Table 3 - Sample tweet dominant emotions identified by different methods*

| Emotion (BERT) | Score BERT | Emotion (Lexicon*) | Text |
| --- | --- | --- | --- |
| Neutral | 0.96 | Neutral | “If there was a Mount Rushmore of NFL linebackers over the past 25 years, Junior Seau would certainly be on it.” |
| Fear | 0.99 | Neutral | “#RIPJuniorSeau Dude was so scary. #SuicidesNotTheAnswer” |
| Surprise | 0.98 | Neutral | “Junior Seau.... I really can't believe that. Super shocked. Prayers out to his family friends and fans. One of the greatest. Rest in peace.” |
| Disgust | 0.99 | Neutral | “Its absolutely disgusting what Junior Seau did. To kill yourself when you have 4 children? What an absolute coward.” |
| Joy | 0.98 | Joy | “I'm glad to hear that numerous people had the foresight to pull out their cell & video Tony Scott climbing an 8' fence jump to his death” |
| Sadness | 0.99 | Neutral | “junior seau's mother speaking about his death is one of the most saddest things I’ve ever watched” |
| Anger | 0.98 | Anger | “Going to rage this weekend in honor of Junior Seau & Adam Yauch. Some great people gone too early.” |
| * Identified using the NRC emotion lexicon approach (https://saifmohammad.com/WebPages/NRC-Emotion-Lexicon.htm) | | | |
