# Supplementary material for: Online propagation of emotions: A study of resharing dynamics on social media following celebrity suicides
Source: PLoS One. 2025 Dec 10;20(12):e0336134. doi: 10.1371/journal.pone.0336134 (PMC12694876; doi:10.1371/journal.pone.0336134)
Supplement: S2 Appendix — (DOCX) [file pone.0336134.s002.docx]

**S2 APPENDIX**

**Validation of Emotion Labels**

To measure the validity of the model-generated labels for predicting the dominant emotions in tweets, we selected a random and balanced sample of tweets in our dataset (n=1000). The sample is balanced using celebrities, such that there is an equal chance for the tweets on each celebrity suicide event to be selected. The sample is annotated by 100 human raters in the US, recruited through Prolific, and these human labels are used as ground truth. Each rater was asked to label 10 randomly selected tweets. Two raters submitted blank responses, so a total of n=980 tweets were used for our evaluation. The DistilRoBERTa model achieved a weighted precision of 0.452, recall of 0.289, and F1 score of 0.346 on our dataset.

**Model Availability**

For our analysis, we use the DistilRoBERTa Transformer model fine-tuned on emotion labeling. Details of the model, including model weights and hyperparameter values, are available online^[[1]](#footnote-2)^. This model has been used in prior work [1] and has previously shown relatively good results in several contexts, including reviews.

We compare the results from the model to that of a baseline classifier where the prediction of labels is weighted random and no learning is involved, i.e., weighted random guess classifier [1]. For all emotional categories, the performance of the model (S2 Table 1) is better than the random guess classifier (S2 Table 2).

S2 Table 1 - Classification performance of DistilRoBERTa, a fine-tuned model for emotion annotation on a subset (N=980) of data.

|  | Anger (1) | Disgust (2) | Fear (3) | Joy (4) | Neutral (5) | Sadness (6) | Surprise (7) | Macro Average | Weighted Average |
| --- | --- | --- | --- | --- | --- | --- | --- | --- | --- |
| Precision | 0.0574 | 0.2 | 0.021 | 0.14 | 0.24 | 0.63 | 0.44 | 0.249 | 0.452 |
| Recall | 0.09 | 0.078 | 0.47 | 0.16 | 0.13 | 0.40 | 0.25 | 0.226 | 0.289 |
| F1-score | 0.070 | 0.11 | 0.041 | 0.15 | 0.17 | 0.49 | 0.32 | 0.193 | 0.346 |
| Support (p) | 22 | 51 | 17 | 43 | 231 | 520 | 96 | 980 | 980 |

S2 Table 2 presents the performance metrics using weighted random guessing on the same subset of tweets for each emotion. In all columns except Anger, we see that the average performance of the model (see S2 Table 1) is better than those using weighted chance guessing (S2 Table 2). We keep all the emotion categories in our subsequent analysis in the manuscript while noting that results for Anger should be interpreted with caution. Cohen’s kappa between model predictions and ground truth was 0.084, and Matthew’s correlation coefficient between model predictions and ground truth was 0.096. These metrics are low but above that of weighted random guessing.

S2 Table 2 - Classification performance of weighted random guessing on the same data subset (N=980)

|  | Anger (1) | Disgust (2) | Fear (3) | Joy (4) | Neutral (5) | Sadness (6) | Surprise (7) | Macro Average | Weighted Average |
| --- | --- | --- | --- | --- | --- | --- | --- | --- | --- |
| Precision | 0.1250 | 0.0400 | 0 | 0.0975 | 0.2394 | 0.5521 | 0.0594 | 0.1590 | 0.3644 |
| Recall | 0.0909 | 0.0392 | 0 | 0.0930 | 0.2467 | 0.5500 | 0.0625 | 0.1546 | 0.3642 |
| F1-score | 0.1052 | 0.0396 | 0 | 0.0952 | 0.2430 | 0.5510 | 0.0609 | 0.1564 | 0.3642 |
| Support (p) | 22 | 51 | 17 | 43 | 231 | 520 | 96 | 980 | 980 |


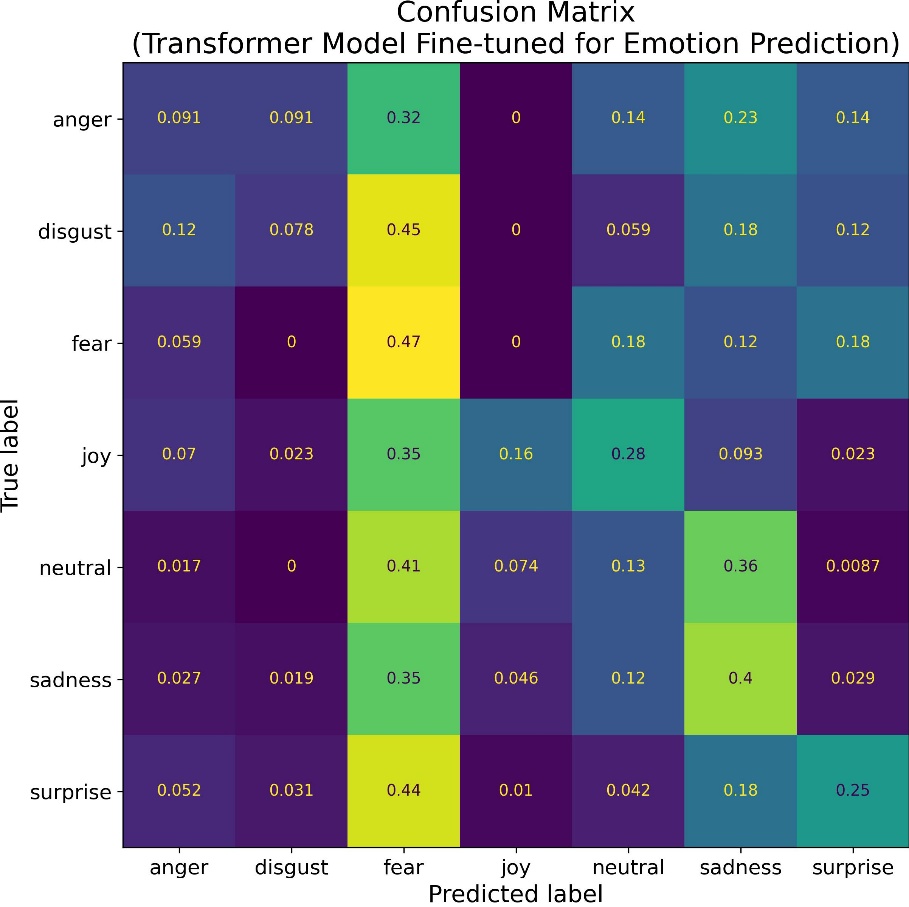


*S2 Figure 1 - The percentage of tweets in each category correctly classified by model are shown in the diagonal cells using human labels as ground truth.*

**References**

1. Rozado D, Hughes R, Halberstadt J. Longitudinal analysis of sentiment and emotion in news media headlines using automated labelling with Transformer language models. Consoli S, editor. PLOS ONE. 2022;17: e0276367. doi:10.1371/journal.pone.0276367

1. <https://huggingface.co/j-hartmann/emotion-english-distilroberta-base> [↑](#footnote-ref-2)
